# Draft Genome Sequences of *Marinobacter similis* A3d10^T^ and *Marinobacter salarius* R9SW1^T^

**DOI:** 10.1128/genomeA.00442-14

**Published:** 2014-05-22

**Authors:** Elena P. Ivanova, Hooi Jun Ng, Hayden K. Webb, Gao Feng, Kenshiro Oshima, Masahira Hattori, Moriya Ohkuma, Alexander F. Sergeev, Valery V. Mikhailov, Russell J. Crawford, Tomoo Sawabe

**Affiliations:** a Swinburne University of Technology, Hawthorn, Victoria, Australia; b Laboratory of Microbiology, Faculty of Fisheries, Hokkaido University, Hakodate, Japan; c Center for Omics and Bioinformatics, Graduate School of Frontier Sciences, University of Tokyo, Kashiwa, Japan; d Microbe Division/Japan Collection of Microorganisms, RIKEN BioResource Center, Ibaraki, Japan; e V. I. Il’ichev Pacific Oceanological Institute of the Far-Eastern Branch of the Russian Academy of Sciences, Vladivostok, Russian Federation; f GB Elyakov Pacific Institute of Bioorganic Chemistry, Far Eastern Branch, Russian Academy of Sciences, Vladivostok, Russian Federation

## Abstract

Here, we present the draft genomes of *Marinobacter similis* A3d10^T^, a potential plastic biodegrader, and *Marinobacter salarius* R9SW1^T^, isolated from radioactive waters. This genomic information will contribute information on the genetic basis of the metabolic pathways for the degradation of both plastic and radionuclides.

## GENOME ANNOUNCEMENT

Gram-negative, aerobic, moderately halophilic gammaproteobacteria with the ability to utilize hydrocarbons as the sole carbon and energy sources were incorporated into the genus *Marinobacter* more than 20 years ago (1). To date, the genus is composed of 33 validly described species (2), with another one yet to be validated (3). Currently, there are seven *Marinobacter* strains that have been reported to have their full genomes sequenced (4–10). Strain A3d10^T^ was isolated from an enrichment experiment selecting for strains that can degrade polyethylene terephthalate (PET) from seawater collected from the first meter below the water surface from St. Kilda Beach, Port Philip Bay, Victoria, Australia (11), while strain R9SW1^T^ was isolated from seawater collected from a radioactive contaminated area in Chazhma Bay, Gulf of Peter the Great, Sea of Japan, Pacific Ocean, Russia (12). In a recent study to screen for potentially PET-degrading marine bacteria, strain A3d10^T^ was found to be able to hydrolyze bis(benzoyloxyethyl) terephthalate (3PET), a PET model substrate (13), whereas strain R9SW1^T^ is of interest for its potential biodegradation of the radionuclides (12). The analyses of the genomes of these two novel *Marinobacter* species will stimulate further research on the metabolite activity, organic pollutant degradation, physiological and ecological functions, and evolution of the bacteria of the genus *Marinobacter*.

On the basis of taxonomic polyphasic analysis, strains A3d10^T^ and R9SW1^T^ are considered to represent novel species of the genus *Marinobacter*, for which the names *Marinobacter similis* A3d10^T^ (type strain A3d10^T^ = JCM 19398^T^) and *Marinobacter salarius* R9SW1^T^ (type strain R9SW1^T^ = JCM 19399^T^ = LMG 27497^T^) are proposed (H. J. Ng, H. K. Webb, D. Gomez, T. Sawabe, J. Ryan, M. Vyssotski, C. Bizet, F. Malherbe, V. V. Mikhailov, R. J. Crawford, E. P. Ivanova, submitted for publication). The genomes of strains A3d10^T^ and R9SW1^T^ were sequenced using an Ion PGM system (Life Technologies, Carlsbad, CA) and *de novo* assembled using the Newbler version 2.8 software. The resulting sequence data for each genome were then submitted to the Microbial Genome Annotation Pipeline (MiGAP) (http://www.migap.org/index.php/en/) (14) and the NCBI Prokaryotic Genomes Automatic Annotation Pipeline (PGAAP) for autoannotation. The open reading frames (ORFs), rRNAs, and tRNAs were also predicted using the MetaGeneAnnotator (MGA) (15), RNAmmer (16), and tRNAscan-SE (17), respectively. The size of the draft genome of strain A3d10^T^ was found to be 3,975,896 bp, composed of 29 contigs, and it has a G+C content of 57.6%. The redundancy is 32, and the *N*_50_ contig length is 386,710 bp. Strain A3d10^T^ contains 3,806 predicted genes, 2,692 putative coding sequences (CDS), 3 rRNAs, and 46 tRNAs. The size of the draft genome of strain R9SW1^T^ was found to be 4,616,532 bp, composed of 99 contigs, and it has a G+C content of 57.1%. The redundancy is 27, and the *N*_50_ contig length is 152,316 bp. Strain R9SW1^T^ contains 4,462 predicted genes, 3,168 CDS, 3 rRNAs, and 44 tRNAs.

### Nucleotide sequence accession numbers.

The genome data have been deposited at GenBank/EMBL/DDBJ under the accession no. CP007151 and CP007152 for *M. similis* A3d10^T^ and *M. salarius* R9SW1^T^, respectively.
